# mSphere of Influence: *Toxoplasma gondii* tissue cysts—who are you calling dormant?

**DOI:** 10.1128/msphere.00028-25

**Published:** 2025-03-12

**Authors:** Robyn S. Kent

**Affiliations:** 1 University of Oklahoma Health Sciences Center, Oklahoma City, Oklahoma, USA; Virginia-Maryland College of Veterinary Medicine, Blacksburg, Virginia, USA

**Keywords:** apicomplexa, parasitology, *Toxoplasma gondii*, latency

## Abstract

Robyn Kent studies how *Toxoplasma gondii* chronic infections can persist in different tissues in the host and how latency is controlled to enable maintenance, transmission, and reactivation of the parasite. In this mSphere of Influence article, she reflects on how two papers from the laboratories of Dr. A. P. Sinai and Dr. L. D. Sibley have impacted her thinking on the chronic stage of *T. gondii* infections.

## COMMENTARY

The life cycles of pathogens are often complex, involving multiple stages in a variety of hosts or vectors. Within these life cycles, we often categorize stages considered chronic, persistent, and less metabolically or physically active as dormant. This classification can downplay the importance and complexity of these stages and discourage investigation into their function and potential for therapeutic targeting. For *Toxoplasma gondii* (*T. gondii*), this “dormant” stage is the tissue cysts that are found in the brain, eyes, heart, and skeletal muscle of hosts. These cysts can transmit the infection to new hosts and can reactivate *in situ* following host immune dysregulation. Reactivation of the chronic infection, to an active infection, is responsible for the most severe clinical manifestations of toxoplasmosis.

As I began researching *T. gondii* as a postdoctoral fellow, I was fascinated by a 2015 *mBio* paper that prompted me to question whether tissue cysts are really dormant. Here, Watts et al. (1) demonstrated that *T. gondii* bradyzoites are dynamic and replicate within tissue cysts that grow *in vivo,* a finding that is at odds with the classic definition of dormancy: “Not active or growing now but able to become active or to grow in the future” (2).

In this work, the authors purify *in vivo* cysts and show that overall cyst burden does not change from chronic infection establishment up to 8 weeks post-infection, but that cysts continue to grow, with a significant increase in size noted at time points generally beyond the standard time point for quantifying and analyzing cysts in the field. They also develop a novel image-processing program, BradyCount 1.0, that allows them to enumerate the individual bradyzoites within the cyst. Using this software, they calculate individual cyst packing density, building upon standard quantifications of cyst number and size. Using this, they found that larger cysts contain more parasites, but are less densely packed than smaller cysts. This outpacing of cyst growth to bradyzoite number demonstrates that parasite replication is not the driving force behind cyst expansion.

In addition, the authors show that bradyzoites within cysts continue to replicate even after encystment and maturation of a chronic infection. Using a marker for the formation of cell progeny formed within the parent cell, the inner membrane complex protein IMC3 that brightly stains progeny and fades as the cell emerges and ages, they found that individual bradyzoites within cysts replicate while others remain inactive and that whole bradyzoite populations can replicate *en masse* within a cyst. These two distinct populations allow for both a rapid (synchronous replication) and slow (asynchronous and clustered replication) increase in bradyzoite number. Taken together, these data show that during the chronic infection, both cysts and bradyzoites are actively increasing in size or number respectively, suggesting that neither cysts nor bradyzoites are truly dormant. They also propose that the maintenance of small cysts shows the re-seeding of new cysts. This seminal work shows that maintaining a chronic *T. gondii* infection is not a dormant process and requires both bradyzoites and cysts to be dynamic.

This group has expanded upon this seminal work to examine the kinetics of bradyzoite replication and begin to establish how the process is powered. They have shown that replicative activity is linked to starch accumulation in the form of amylopectin granules, amylopectin granule degradation, and the metabolic activity of the bradyzoites within the cysts (3, 4).

Additionally, another group has recently demonstrated that the composition of the cyst wall is dynamic and the constituent proteins making up the wall must be carefully maintained to allow the formation of progeny cysts, which is critical for long-term cyst persistence. Using a chitinase-like protein (CLP1) knockout, they show that in the absence of CLP1, the cyst wall becomes thicker, and the protein composition alters with several proteins over or underrepresented following knockout of CLP1. This alteration in wall composition prevents the formation of cyst progeny *in vitro*, suggesting that the cyst wall becomes too thick or rigid to allow cyst budding or re-seeding of cysts. The thickening caused by CLP1 deletion is also associated with a reduction in cyst burden over time *in vivo* (5).

These works show that the *T. gondii* chronic infection is more than just a dormant cyst waiting to become something more interesting. Begging the question, if the parasites and the cysts are not dormant, what are they doing? To me, this also renews my optimism that we can target the chronic stage to cure these infections.

## References

[1] Watts E, Zhao Y, Dhara A, Eller B, Patwardhan A, Sinai AP. 2015. Novel approaches reveal that Toxoplasma gondii bradyzoites within tissue cysts are dynamic and replicating entities in vivo. mBio 6:e01155-15. doi:10.1128/mBio.01155-15 26350965 PMC4600105

[2] Stevenson A, Waite M. 2011. Concise Oxford English dictionary. 12th ed, p 1696. Oxford University Press, Oxford, United Kingdom.

[3] Murphy RD, Troublefield CA, Miracle JS, Young LEA, Tripathi A, Brizzee CO, Dhara A, Patwardhan A, Sun RC, Kooi CWV, Gentry MS, Sinai AP. 2024. TgLaforin, a glucan phosphatase, reveals the dynamic role of storage polysaccharides in Toxoplasma gondii tachyzoites and bradyzoites. bioRxiv:2023.09.29.560185. doi:10.1101/2023.09.29.560185

[4] Tripathi A, Donkin RW, Miracle JS, Murphy RD, Gentry MS, Patwardhan A, Sinai AP. 2024. Dynamics of amylopectin granule accumulation during the course of the chronic Toxoplasma infection is linked to intra-cyst bradyzoite replication. bioRxiv:2024.09.02.610794. doi:10.1101/2024.09.02.610794 PMC12306163 40492719

[5] Fu Y, Tomita T, Weiss LM, West CM, Sibley LD. 2025. Toxoplasma chitinase-like protein orchestrates cyst wall glycosylation to facilitate effector export and cyst turnover. Proc Natl Acad Sci USA 122:e2416870122. doi:10.1073/pnas.2416870122 39879244 PMC11804682

